# Hot melt extruded-based nano zinc as an alternative to the pharmacological dose of ZnO in weanling piglets

**DOI:** 10.5713/ajas.19.0140

**Published:** 2019-10-21

**Authors:** Seung Min Oh, Min Ju Kim, Abdolreza Hosseindoust, Kwang Yeol Kim, Yo Han Choi, Hyung Bin Ham, Sung Jun Hwang, Jun Hyung Lee, Hyun Jong Cho, Wei Soo Kang, Byung Jo Chae

**Affiliations:** 1 Gyeongbuk Livestock Research Institute, Yeongju 63052, Korea; 2 Department of Animal Resources Science, College of Animal Life Sciences, Kangwon National University, Chuncheon 24341, Korea; 3 Swine Division, National Institute of Animal Science, Rural Development Administration, Cheonan 31000, Korea; 4 College of pharmacy, Kangwon National University, Chuncheon 24341, Korea; 5 Department of Bio-Health Technology, College of Bio-Medical Science, Kangwon National University, Chuncheon 24341, Korea

**Keywords:** Nano Zinc, Villus Height, Microbiota, Weaned Pigs, Bioavailability

## Abstract

**Objective:**

This study was conducted to investigate the effects of hot-melt extruded ZnO nano-particles (HME-ZnO) as an alternative for P-ZnO on growth performance, nutrient digestibility, Zn bioavailability, intestinal microbiota, and intestinal morphology of weanling pigs.

**Methods:**

A total of 450 piglets (Landrace×Yorkshire×Duroc) were randomly allotted to five treatments based on initial body weight and sex. The experimental diets were fed in a meal form as phase 1 from d 0 to 14 and phase 2 from d 15 to 28. Treatments were the control diet without ZnO supplementation, the diet containing 2,500 ppm Zn as ZnO, and three diets containing 500, 1,000, or 2,500 ppm Zn as HME-ZnO.

**Results:**

The overall result showed a higher (p<0.01) average daily gain in weanling pigs fed ZnO-supplemented diets in comparison to the control diet. There was a decrease (p<0.01) in fecal score in the ZnO-supplemented diets. Dietary supplementation of ZnO improved (p<0.05) crude protein digestibility. The weanling pigs fed the P-ZnO diet had a lower (p< 0.01) Zn digestibility in the feces than HME-ZnO supplemented treatments. Weanling pigs fed diets supplemented with ZnO had greater (p<0.05) *Lactobacillus* spp. populations and lower *Clostridium* spp. (p<0.05) and Coliforms (p<0.01) populations in the ileum. Weanling pigs fed diets supplemented with increasing concentrations of HME-ZnO linearly decreased *Clostridium* spp. (p<0.05) and Coliforms (p<0.01) in the ileum. Lower (p<0.05) *Clostridium* spp. and Coliforms counts in the colon were observed in pigs fed with ZnO-supplemented diets. Weanling pigs fed diets supplemented with ZnO showed a greater (p<0.01) villus height in the duodenum.

**Conclusion:**

Dietary supplementation of HME-ZnO and P-ZnO showed a potential to improve the digestibility of protein, intestinal Coliform and *Clostridium*, villus height in duodenum and ileum. Moreover, HME-ZnO showed a higher Zn digestibility compared with P-ZnO.

## INTRODUCTION

Diarrhea is a critical stressor in weanling pigs, which often leads to growth depression and post-weaning diarrheic syndrome [1–3]. The high susceptibility to enterotoxigenic *Escherchia coli* (*E. coli*) and *Clostridium perfringens* as the most common infectious agents during weaning may increase the incidence of diarrhea and decrease growth of weanling pigs [1,4,5]. In recent years, research has been conducted to find non-antibiotic feed additives, which have the potential to improve gut health and performance of weanling pigs [6,7]. Particularly, after the ban on the utilization of antibiotics in livestock diets, dietary ZnO supplementation at the pharmacological dose (2,500 and 3,000 mg Zn/kg) has been widely used to improve the growth performance and gastrointestinal functions and to markedly alleviate diarrhea in young pigs [6]. However, regarding environmental concerns, many countries in European Union [8] and other countries such as South Korea or Japan legislated the restricted use of ZnO.

Recently, nano-particles have been widely used in food, medicine, and pharmaceutical industries as more active options with higher biological potential than conventional sources due to their smaller size and larger surface area [9]. In simple terms, the particle size of 1 to 100 nm is defined as nano minerals [9]. In addition, the methods of nano-particle production may affect their biochemical properties as well. Hot melt extrusion (HME) is a continual mixing process applied to melt or solubilize ingredients and medicines by using a mechanical shear inside the matrix of polymer to produce amorphous systems, which improves solubility and increases the bioavailability of nano-particles [9]. To our knowledge, this study is the first report on the bioavailability of nano-ZnO as HME. In this study, Soluplus (BASF, Ludwigshafen, Germany) was applied as the main polymer matrix during HME processing. The amphiphilic properties of Soluplus optimize solubility and bioavailability through the polyethylene glycol, polyvinyl caprolactam, and polyvinyl acetate contents [9]. Soluplus is a copolymer with the capability of deploying as a binder in wet or dry granulation to increase the solubility and dispersion of poorly water-soluble drugs [9]. The Soluplus-included HME manufacturing process can be applied in the animal feed industry to decrease the environmental pollutions through the reduced trace mineral excretion. Therefore, the effects of dietary supplemented ZnO and hot melt extruded ZnO (HME-ZnO) sources were evaluated on growth performance, nutrient digestibility, intestinal morphology, and microbiota in weanling pigs.

## MATERIALS AND METHODS

The experiment was conducted at the facility of Kangwon National University farm and was approved by the Institutional Animal Care and Use Committee of Kangwon National University, Chuncheon, Republic of Korea.

### Preparation of ZnO and HME-ZnO

Zinc oxide was purchased from TMC Co., Ltd. (Anyang, Korea). Zinc oxide nano-particles were designed and produced using HME techniques by maintaining optimum processing conditions. The production assay was carried out as suggested in an analytical procedure [9]. In short, the optimum conditions during HME operations were: temperature, 50°C to 55°C; speed of screw, 150 rpm; diameter of die, 1.0 mm; production rate, 3 kg/h. Soluplus (grafted copolymer) and ZnO were mixed in the proper ratio (7:3, w/w) and then extruded by a twin-screw hot-melt extruder (STS-25HS, Hankook E.M. Ltd., Pyeongtaek, Korea) equipped with a round-shaped die (2 mm diameter) to produce the final product of HME-ZnO. The speed of the screw was set at 200 rpm and extrudates were cooled and milled by the HBL-3500S grinder (Samyang Electronics Co., Gunpo, Korea) for pulverization.

### Animals, diets, and managements

A total of 450 piglets (Landrace×Yorkshire×Duroc) with average body weight (BW) of 6.86±0.3 kg were allotted to five treatments on the basis of initial BW and sex (all male or all female) in a randomized complete block design. There were six replicate pens in each treatment with 15 pigs per pen. Dietary treatments consisted of the control diet without ZnO supplementation, the diet containing 2,500 ppm Zn as ZnO, and three diets containing 500, 1,000, or 2,500 ppm Zn as HME-ZnO (Table 1). Chromic oxide (2.50 g/kg) was added in each diet. Corn was replaced by ZnO sources based on the ZnO supplementation level in the diet. The experimental diets exceeded the nutrient requirements as suggested by the NRC [10], and were fed in a meal form for two phases (phase 1 from d 0 to 14 and phase 2 from d 15 to 28). The pigs used in the study were housed in partially slotted concrete floor pens with pens with a size of 2.80 m×5.00 m. All the pens were equipped with a feeder and a nipple drinker to allow *ad libitum* access to feed and water.

### Growth performance and nutrient digestibility

At the end of each phase, pigs were weighed individually, and feed consumption was measured to calculate average daily gain (ADG), average daily feed intake (ADFI), and gain to feed ratio (G:F). Fecal grab samples were collected during the last 4 d of each phase to determine the apparent total tract digestibility of dry matter (DM), gross energy (GE), crude protein (CP), and Zn. Pens were cleaned before the start of sample collection and the fecal samples were then pooled within the pen, dried in a forced air oven at 60°C for 72 h, and ground in a Wiley mill (Thomas Model 4 Wiley Mill, Thomas Scientific, Swedesboro, NJ, USA) using a 1-mm screen for chemical analysis. Analysis for each sample was done in triplicate for DM (Method 930.15), CP (Method 990.03), ash (Method 942.05), Ca, and P (method 985.01; 16) according to the methods of AOAC [11]. The GE of diets and feces were measured using a bomb calorimeter (Model 1261, Parr Instrument Co., Molin, IL, USA), while Cr concentrations were determined [12] with an automated spectrophotometer (Shimadzu, Japan).

### Determination of Zn contents

Zinc content (method 985.01) in the feed, feces, and serum was determined on the dissolved ashes prepared by AOAC [11]. One g of ground feed or fecal sample was ashed for 1 h in a muffle furnace at 600°C. Then, the ashed sample was allowed to cool, dissolved by adding 10 mL 50% HCl (v/v), and kept covered overnight. For serum samples, a 1 mL serum sample was measured in porcelain crucible and dried for 4 hours at 105°C and then ashed for 1 h at 600°C in a muffle furnace. The samples were filtered using Whatman filter paper No.40 (Whatman, Maidstone, UK) in a 100 mL volumetric flask by washing crucibles 2 to 3 times and diluted with deionized distilled water. The concentration of Zn was measured by inductively coupled plasma emission spectroscopy (730-ES, Varian, Palo Alto, CA, USA).

### Blood collection and immunoglobulin analysis

A 10 mL blood sample was collected from two randomly selected pigs from each pen on d 14 and 28. The sampling was conducted by jugular vein puncture using a disposable vacutainer tube without anticoagulants (Becton Dickinson, Franklin, NJ, USA). The blood samples were then centrifuged by 3,000×*g* for 15 min 4°C, and separated serum was stored at −20°C until analysis. Immunoglobulin A (IgA), IgG, and IgM of serum were analyzed with immunoglobulin kits (Sanwei Biological Engineering Co., Ltd., Shandong, China), using an automated spectrophotometer (Shimadzu, Japan).

### Microbial analyses

To evaluate the microbial population, on d 28, fresh digesta samples of ileum, cecum, and colon from 2 euthanized pigs (around the average BW) in each pen were collected and kept on ice. One gram of the digesta from ileum, cecum, or colon was diluted with 9 mL of 1% peptone broth (Becton, Dickinson and Co., USA) and then homogenized. Viable counts of bacteria in the samples were then conducted by plating serial 10-fold dilutions to determine the total anaerobic bacteria (tryptic soy agar), *Lactobacillus* spp. (De Man, Rogosa and Sharpe agar) + 0.2 g/L NaN3 + 0.5 g/L L-cystine hydrochloride monohydrate), *Clostridium* spp. (tryptose sulfite cycloserine [TSC] agar), and Coliform (violet red bile agar) were used. The anaerobic conditions during the assay of total anaerobic bacteria and *Clostridium* spp. were created by using gas pack anaerobic system (BBL, No. 260678, Difco, Detroit, MI, USA). The tryptic soy agar (No. 236950) and violet red bile agar (No. 216695) were purchased from Difco Laboratories (USA), and TSC agar (CM0589) were purchased from Oxoid (Hampshire, UK). The bacterial concentrations were transformed to log before statistical analysis.

### Small intestinal morphology

To evaluate the small intestinal villus height and crypt depth, on d 28, intestinal samples from euthanized pigs (1 male and 1 female) in each pen were collected. For each intestinal sample, three cross-sections were prepared after staining with azure A and eosin using standard paraffin embedding procedures [13]. A total of 10 intact, well-oriented crypt-villus units were selected in triplicate for each intestinal cross-section. The measurement of villus height was made from the tip of the villi to the villus-crypt junction, the crypt depth was defined as the depth of the invagination between adjacent villi, and villus width was measured till the middle of the villus. All morphological measurements were made in 10-μm increments by using an image processing and analysis system (Optimus software version 6.5, Media Cybergenetics, North Reading, MA, USA).

### Calculations and statistical analysis

The apparent total tract digestibility was calculated as:

$$\text{The apparent total tract digestibility}=1-({\text{Cr}}_{\text{diet}}\times {\text{nutrient}}_{\text{feces}})/({\text{Cr}}_{\text{feces}}\times {\text{nutrient}}_{\text{diet}})$$

where, Cr_diet_, chromium content in the diet; nutrient_feces_, nutrient content in the feces; Cr_feces_, chromium content in the feces; nutrient_diet_, nutrient content in the diet.

Orthogonal contrasts (control vs others; HME-ZnO vs P-ZnO) were used to compare the possible relationship between the treatments, using the procedure PROC general linear model of SAS (SAS Inst. Inc., Cary, NC, USA). In addition, orthogonal polynomial contrasts were applied to evaluate the significance of linear or quadratic models to describe the response in increasing dietary HME-ZnO levels (0, 500, 1,000, and 1,500 mg/kg). The pen was used as the experimental unit for the analysis of all the parameters. Probability values less than 0.05 were considered as significance.

## RESULTS

### Growth performance and fecal score

Growth performance and fecal score data are presented in Table 2. There was no mortality in this experiment (data not shown). In phase 1, there was an improvement (p<0.01) in ADG and G:F in pigs fed ZnO-supplemented diets compared with the control diet. However, there was no difference in ADG, ADFI, and G:F between HME-ZnO and P-ZnO treatments. An increasing concentration of HME-ZnO in diets resulted in linearly improved (p<0.01) ADG. In phase 2, Dietary supplementation of ZnO showed an increased (p<0.01) ADG and G:F compared with the control diet. Weanling pigs fed diets supplemented with increasing concentrations of HME-ZnO increased ADG (linear and quadratic, p<0.01), and G:F (linear, p<0.01). The overall result showed a greater (p<0.01) ADG and G:F in weanling pigs fed ZnO-supplemented diets in comparison to the control diet. There was no difference in ADG, ADFI, and G:F between the HME-ZnO and P-ZnO diets. Weanling pigs fed the HME-ZnO diets showed a greater ADG (linear and quadratic, p<0.05), ADFI (linear, p<0.05), and G:F ratio (linear, p<0.01) during overall phase. In phases 1 and 2, there was a decrease (p<0.01) in fecal score in the ZnO-supplemented diets. Weanling pigs fed P-ZnO had a lower fecal score (p<0.01) compared with the HME-ZnO treatments. The HME-supplemented diets showed reduced fecal score in phase 1 (linear and quadratic, p<0.05) and phase 2 (linear, p<0.05).

### Digestibility of nutrients

The effects of dietary supplemented Zn source and concentration on nutrient digestibility are shown in Table 3. In phase 1, there were no differences in digestibility of DM and GE. Dietary supplementation of ZnO improved (p<0.05) CP digestibility compared with the control treatment. However, there was no difference in digestibility of CP between the HME-ZnO and P-ZnO treatments. There was a linear increase (p<0.01) of CP digestibility by the supplementation of HME-ZnO to the diet. In phase 2, there was no difference in digestibility of DM. Weanling pigs fed ZnO-supplemented diets showed a greater digestibility of GE (p<0.05) and CP (p<0.01) compared with weanling pigs fed the control diet. There was a linear increase (p<0.01) in digestibility of GE and CP as the dietary HME-ZnO inclusion level increased.

### Immunoglobulins

IgG, IgA, and IgM levels were not affected in weanling pigs fed diets supplemented with ZnO sources in phase 1 and 2 (Table 4).

### Zn concentrations in feces and serum

There was an increase (p<0.01) in the concentration of Zn in feces with ZnO supplementation in the diets in both phases (Table 5). The weanling pigs fed the P-ZnO diet had a greater (p<0.01) Zn concentration in the feces and lower (p<0.01) Zn digestibility than HME-ZnO supplemented treatments. In phases 1 and 2, an increasing concentration of HME-ZnO in the diets resulted in linear and quadratic increase (p<0.01) in Zn concentration in the feces and linear increase (p<0.01) in Zn digestibility. Weanling pigs fed the control diet showed a lower (p<0.01) concentration of Zn in serum compared with ZnO-supplemented treatments in both phases. However, there were no differences in serum Zn concentration between P-ZnO and HME-ZnO treatments. An increasing concentration of HME-ZnO in the diets resulted in linear (phases 1 and 2) and quadratic (phase 1) increase (p<0.01) in Zn concentration in serum.

### Intestinal microbiota

Effects of dietary supplemented Zn source and concentration on intestinal microbiota are shown in Table 6. No difference was detected in the population of total anaerobic bacteria in the ileum. Weanling pigs fed diets supplemented with ZnO had greater (p<0.01) *Lactobacillus* spp. populations and lower *Clostridium* spp. and Coliforms populations in the ileum compared with the control. Weanling pigs fed diets supplemented with increasing concentrations of HME-ZnO decreased *Clostridium* spp. (linear and quadratic, p<0.05) and Coliforms (linear, p<0.01) in the ileum. There were no differences in *Lactobacillus* spp., *Clostridium* spp., and Coliforms population in the cecum between pigs fed the ZnO-supplemented diets and the control diet, and also between HME-supplemented and P-ZnO treatments. In the cecum, *Clostridium* spp. population decreased (linear, p<0.01) with a dietary increasing concentration of HME-ZnO. Lower (p<0.05) *Clostridium* spp. and Coliforms counts in colon were observed in weanling pigs fed with ZnO-supplemented diets than that of pigs fed the control diet. The counts of *Clostridium* spp. in cecum and colon were linearly decreased (p<0.01) in weanling pigs fed HME-ZnO diets.

### Intestinal morphology

Effects of dietary supplemented Zn source and concentration on small intestinal morphology are shown in Table 7. Weanling pigs fed diets supplemented with ZnO showed a greater (p<0.01) villus height and villus height to crypt depth ratio (VH/CD) in the duodenum. Weanling pigs fed diets supplemented with increasing concentrations of HME-ZnO increased villus height (linear and quadratic, p<0.05) and VH/CD (linear, p<0.01) in the duodenum. Weanling pigs diets supplemented with ZnO increased (p<0.05) VH/CD in the jejunum compared with weanling pigs fed the control diet. There was no difference in villus height and crypt depth in the jejunum between pigs fed P-ZnO and HME-ZnO diets. In the jejunum, an increasing concentration of HME-ZnO in diets resulted in linearly improved crypt depth and VH/CD (p< 0.05). A greater (p<0.05) villus height in the ileum was observed in weanling pigs fed ZnO supplemented diets. There was a linear increase (p<0.01) in the villus height of the ileum as the HME-ZnO inclusion level increased.

## DISCUSSION

The greater overall ADG in weanling pigs fed ZnO-supplemented diets could be explained in part by the improved digestibility of nutrients. In addition, the pathogens suppression in the commensal gut microbiota in the intestine, besides a potential promotion of intestinal morphology, may further render nutrients available for weaned pigs by high dietary ZnO doses supplementation. There are two possible hypotheses that may justify the significant role of ZnO in improving the growth performance of weanling pigs. Firstly, Zn is a well-known anti-stress element and the positive effects on ADG may be explained by the antioxidative role of Zn as a main contributor to antioxidant enzymes production [14]. Especially that weaning stress is aligned with a high lipid peroxidation and a reduced glutathione peroxidase activity [14]. Therefore, it seems that Zn requirement can be much higher than the other growing stages and a higher Zn absorption may trigger the antioxidant capacity of weanling piglets. Secondly, ZnO has been frequently used as an antimicrobial agent in intestine due to its direct effects on pathogens such as *E. Coli* [5]. Un-absorbed ZnO releases to distal sections of intestine and reduce the colonization of pathogens [5,15]. Apart from the concentration of ZnO in the diet, the particle size of ZnO may be a crucial factor in determining the extent of influence on pathogens. The ZnO nanoparticle includes high surface area and more active surface than those of a regular size [16–18]. Thus, even a lower dose of nano-sized ZnO may show a similar anti-pathogenic activity as a high dose of ZnO in regular form. The greater ADG is in agreement with previous studies which reported that diets containing pharmacological concentration of Zn as ZnO led to increased ADG in weanling pigs [19,20]. It was reported that the concentration of dietary Zn at 2,000 ppm as ZnO enhanced the growth performance of weaned pigs, whereas lower doses at 500 or 1,000 ppm were not effective [21]. This agrees with the results of the present study, where the supplementations of HME-ZnO in pigs diet showed a linear increase in ADG. Previous studies reported that the high dietary Zn doses (e.g., 2,000 to 3,000 ppm Zn as ZnO) improved growth performance or feed efficiency in pigs [16,19,20]. However, in other studies, a high dose of ZnO had no effect on growth performance [2,3,5,17]. The first phase after weaning is the most critical period for pigs mainly because of a sudden change in the diet from milk to solid. However, the greater ADG and G:F in pig fed ZnO-supplemented diets in the second phase emphasize the importance of using the high doses of ZnO in the phase 2 as well. The results of this study also suggested that using sub-pharmacological dose of HME-ZnO shows no difference in ADG when compared with regular P-ZnO. It also may imply that reducing the dose of ZnO as HME can be still effective.

Fecal Zn concentration was greater in pigs fed ZnO-supplemented diets compared with the control. The concentration of Zn in the feces of weaning pigs fed the diets supplemented with 1,750 or 3,500 mg/kg of Zn was consistently greater than that pigs fed the control diet [19]. It was also reported that around 20-fold increase in Zn excretion has been observed as dietary Zn increased from 0 to 3,000 mg/kg [17,22]. In the present study, HME-ZnO was absorbed better than P-ZnO, which is consistent with the finding of Li et al [18] that ZnO digestibility in nanoparticle form was 56% higher than the conventional ZnO. Nanoparticles have been reported to show a greater absorption rate in the intestine by penetrating small capillaries and beingtaken up by enterocytes [9,17]. Our study also has demonstrated that reducing Zn particles to nanosize increases their intestinal uptake. Nano-ZnO possesses lower particle size and larger number of particles per unit, resulting in higher digestibility. High digestibility of Zn can decrease Zn excretion, consequently reducing the environmental issues. Moreover, the linearly increased serum Zn level in HME-ZnO treatments is in agreement with Han et al [6], who reported an increase in serum Zn concentration when pigs fed 1,500 or 2,500 ppm as ZnO. Pieper et al [15] reported that the serum Zn concentration of pigs fed diets including different levels of ZnO was the lowest in diets containing 50 mg/kg Zn, intermediate in diets containing 150 or 250 mg/kg Zn, and the greatest in diets containing 1,000 or 2,500 mg/kg Zn. Despite a greater digestibility, the serum Zn concentration was not different between ZnO-HME and P-ZnO treatments. In this study, we did not evaluate the concentration of Zn in the liver and kidney, however, the insignificant differences in the serum Zn concentration between the HME-ZnO and inorganic-ZnO treatments may emphasize the role of storage organs. Plasma Zn concentrations reach a saturation point after using of pharmacological doses of ZnO in weanling piglets for two weeks and to some extent the excess plasma Zn renders to the liver and kidney to be stored [23].

In the present trial, pigs fed diets supplemented with ZnO had greater *Lactobacillus* spp. in the ileum than pigs fed the control diets, which is in contrast to previous findings [15,17]. In addition, the counts of Coliforms and *Clostridium* spp. in the ileum of pigs fed ZnO diets were lower than pigs fed the control diet. A 2,500 ppm ZnO/kg diet needs to be supplemented into the diet of weanling pigs to exert anti-diarrhea effects [5]. Even when nano-ZnO are used, the dietary dosage of ZnO for diarrhea control is much higher [17] than dietary Zn recommendations for requirement in standard references [10]. The antibacterial specification of ZnO is associated with the hydrogen peroxide generation from its surface [24]. Hydrogen peroxide generation may gradually decrease the intestinal pH. A more acidic milieu prevents overgrowth of pathogenic bacteria, such as *E. coli*, while *Lactobacillus* spp. can tolerate an acidic environment [25]. The result of this study shows that ZnO mainly decreases the pathogen (Coliform and *Clostridium*) populations than *Lactobacillus* spp. Therefore, it is assumed that ZnO has the ability to limit pathogen growth and indirectly gives an opportunity for *Lactobacillus* spp. to proliferate in a less competitive environment. In contrast, another study showed no additional benefits of using ZnO on ileal *Lactobacillus* spp. populations, but reported greater ileal total anaerobic bacteria and lower Coliforms population [5]. It has been well established that ZnO beneficially affects weaned pigs by improving the balance of microbial populations that suppress pathogens like Coliforms [2,20]. Another experiment with weaned pigs showed that the number of clostridial clusters were reduced at the pharmacological dose of Zn at 2,500 ppm as ZnO was without any effects on *Clostridium* spp. population when lowering the doses of Zn to 50, 150, 250, and 1,000 ppm, most likely reflecting a decreased pathogenic bacteria in the ileum of weanling pigs receiving the high Zn level [15]. The population of Coliforms and *Clostridium* spp. in the ileum were linearly decreased by increasing dietary HME-ZnO supplementation. The increase of ZnO surface area by decreasing particle size can increase the antibacterial activity by efficient H_2_O_2_ generation [24]. Yamamoto [26] reported that the decrease in particle size of ZnO increase the antibacterial activity of ZnO. The result of this study indicates that the sub-pharmacological dose of HME-ZnO as well as P-ZnO are effective in alleviating pathogen colonization in the proximal sections gastrointestinal tract of weaning piglets.

Post-weaning stress causes transient crypt hyperplasia and villus atrophy during weaning [1,7]. The present study indicated that villus height in the duodenum and ileum was improved in ZnO-supplemented diets. Hosseindoust et al [5] demonstrated an increase in dietary ZnO improved villus height in the duodenum, which might lead to an increased nutrient digestibility in weanling pigs and suggested that ZnO may affect the morphology and function of the intestine in weanling pigs, resulting in a temporary increase in the intestine surface area by increasing the villus size. Moreover, our results demonstrated that the supplementation of ZnO into the diet increased the digestibility of CP. Intestinal morphology is an indicator of nutrient digestion, as pigs fed HME-ZnO diets showed a linear increase in digestibility of CP and villus height in the duodenum and ileum. This is in line with the earlier study where digestibility of DM and jejunal villus height were improved in weaned pigs supplemented with the pharmacological dose of ZnO [2,20]. The increase in the digestibility may have been due to improvement in intestinal morphology. This could be beneficial for the weaned pigs because improved intestinal morphology explains proper and rapid absorption of nutrients.

In conclusion, dietary supplementation of HME-ZnO and P-ZnO improved the performance of weaned pigs, which may be associated with the regulated composition of the gut microflora, and improved digestibility of CP and intestinal villus height of piglets. In addition, dietary supplementation with sub-pharmacological dose of HME-ZnO may be an alternative to the use of P-ZnO due to the insignificant differences in performance and lower Zn excretion. However, further studies are needed to indicate what dose of HME-ZnO is the best recommendation for weanling piglets.

## Figures and Tables

**Table 1:** Formula and chemical composition of control diets (as-fed basis)

Item	Phase 1 (d 0 to 14)	Phase 2 (d 15 to 28)
Ingredient (g/kg)		
Corn	380.7	465.7
Fish meal	50	30
Dehulled soybean meal	244.9	294.9
Whey powder	170	153.8
Fish meal	50	30
Soy protein concentrate	50	-
Soybean oil	33.8	26
Monocalcium phosphate	4	3.6
Limestone	7.7	8.3
Salt	3	3
DL-methionine	1.4	0.8
L-lysine	3.3	2.8
L-threonine	1.4	1.3
L-tryptophan	2.2	1.7
Vitamin premix1)	2.5	2.5
Mineral premix2)	2.5	2.5
Choline chloride	0.5	0.5
Phytase	0.1	0.1
Chromic oxide	2.5	2.5
Lactose	39.5	-
Total	1,000	1,000
Calculated composition (%)		
ME (MJ/kg)	14.226	14.226
CP	23	21
Calcium	0.8	0.7
Available phosphorus	0.4	0.33
SID Lys	1.35	1.23
SID Met	0.41	0.36
SID Met+Cys	0.74	0.68
SID Thr	0.79	0.73
SID Trp	0.22	0.2
Lactose	15	10

ME, metabolizable energy; CP, crude protein; SID, standardized ileal digestibility

1) Supplied per kilogram of diet: 20,000 IU vitamin A, 4,200 IU vitamin D_3_, 10 IU vitamin E, 5.6 mg vitamin K_3_, 2.8 mg vitamin B_1_, 5.5 mg vitamin B_2_, 4.2 mg vitamin B_6_, 0.042 mg vitamin B_12_, 14 mg pantothenic acid, 42 mg niacin, 0.105 mg biotin, 1.05 mg folic acid.

2) Supplied per kilogram of diet: 50 mg Fe as FeSO_4_, 0.20 mg Co as CoSO_4_, 30 mg Cu as CuSO_4_, 30 mg Mn as MnSO_4_, 0.35 mg I as Ca(IO_3_), 0.25 mg Se as Na_2_SeO_3_.

**Table 2:** Effects of different ZnO sources on growth performance and fecal score in weanling pigs

Item	CL1)	HME1) (ppm)			P-ZnO1)	SEM	Contrasts2) (p-value)			
	
		500	1,000	2,500			CL vs others	HME vs P-ZnO	L	Q
Phase 1 (0–14 d)										
ADG (g)	255	261	281	289	285	5.58	0.009	0.293	<0.001	0.888
ADFI (g)	379	377	393	402	395	10.62	0.316	0.706	0.088	0.599
G:F	0.67	0.70	0.71	0.72	0.72	0.01	0.004	0.456	0.700	0.419
Phase 2 (15–28 d)										
ADG (g)	399	442	443	435	448	5.32	<0.001	0.229	<0.001	<0.001
ADFI (g)	623	635	658	644	657	11.68	0.065	0.391	0.073	0.195
G:F	0.64	0.66	0.67	0.67	0.68	0.01	<0.001	0.042	<0.001	0.074
Overall (0–28 d)										
ADG (g)	327	351	362	362	367	4.32	<0.001	0.137	<0.001	0.031
ADFI (g)	501	506	526	523	526	8.21	0.078	0.432	0.041	0.673
G:F	0.66	0.68	0.69	0.69	0.7	0.01	<0.001	0.103	<0.001	0.180
Fecal score3)										
Phase 1	3.81	2.84	3.09	2.79	2.63	0.15	<0.001	0.006	<0.001	0.011
Phase 2	2.69	2.34	1.88	1.72	1.39	0.10	<0.001	<0.001	<0.001	0.384

HME, hot melt extrusion; SEM, standard error of means; ADG, average daily gain; ADFI, average daily feed intake; G:F, gain to feed ratio.

1) CL, basal diet without ZnO; HME, basal diet with 500, 1,000, and 2,500 ppm Zn as HME-ZnO; P-ZnO, Basal diet with 2,500 ppm Zn as ZnO.

2) Linear (L) and quadratic (Q) effects of HME concentrations.

3) 1 = hard feces, 2 = firm well formed, 3 = soft and partially formed feces, 4 = loose, semi-liquid feces, and 5 = watery feces.

**Table 3:** Effects of different ZnO sources on apparent total tract digestibility (%) of nutrients in weanling pigs

Item	CL1)	HME1) (ppm)			P-ZnO1)	SEM	Contrasts2) (p-value)			
	
		500	1,000	2,500			CL vs others	HME vs P-ZnO	L	Q
Phase I (d 14)										
Dry matter	82.3	82.5	82.7	82.6	83.4	0.73	0.549	0.449	0.723	0.759
Gross energy	80.1	80.7	81.1	80.7	81.7	0.43	0.096	0.093	0.217	0.222
Crude protein	76.4	76.6	78	77.9	78.2	0.45	0.012	0.161	0.005	0.677
Phase II (d 28)										
Dry matter	81	81.1	81.9	81.6	82.5	0.44	0.219	0.147	0.133	0.645
Gross energy	79.8	80.1	80.8	80.9	81.2	0.38	0.044	0.211	<0.001	0.797
Crude protein	75.6	77.4	77.7	78.1	77.2	0.43	0.002	0.347	<0.001	0.063

HME, hot melt extrusion; SEM, standard error of means.

1) CL, basal diet without ZnO; HME, basal diet with 500, 1,000, and 2,500 ppm Zn as HME-ZnO; P-ZnO, basal diet with 2,500 ppm Zn as ZnO.

2) Linear (L) and quadratic (Q) effects of HME concentrations.

**Table 4:** Effects of different ZnO sources on the serum immunoglobulins (mg/dL) in weanling pigs

Item	CL1)	HME (ppm)			P-ZnO1)	SEM	Contrasts2) (p-value)			
	
		500	1,000	2,500			CL vs others	HME vs P-ZnO	L	Q
Phase I (d 14)										
IgG	249	238	225	232	236	15.5	0.652	0.811	0.321	0192
IgA	27.2	27.5	28.7	28.6	29.5	0.57	0.077	0.112	0.112	0526
IgM	6.25	6.75	6.67	6.75	6.50	0.27	0.185	0.451	0.185	0.623
Phase II (d 28)										
IgG	264	248	242	261	245	14.9	0.319	0.725	0.912	0.348
IgA	27.9	28.9	29.7	28.6	30.2	0.53	0.087	0.259	0.522	0.205
IgM	6.50	7.25	7.00	6.88	6.75	0.22	0.126	0.264	0.698	0.126

HME, hot melt extrusion; SEM, standard error of means; Ig, immunoglobulin.

1) CL, basal diet without ZnO; HME, basal diet with 500, 1,000, and 2,500 ppm Zn as HME-ZnO; P-ZnO, basal diet with 2,500 ppm Zn as ZnO.

2) Linear (L) and quadratic (Q) effects of HME concentrations.

**Table 5:** Effects of different ZnO sources on Zn concentration of feces and serum in weanling pigs

Item	CL1)	HME1) (ppm)			P-ZnO1)	SEM	Contrasts2) (p-value)			
	
		500	1,000	2,500			CL vs others	HME vs P-ZnO	L	Q
Phase I (d 14)										
Feed (mg/kg DM)	179	721	1,219	2,688	2,694	-	-	-	-	-
Feces (μg/g)	1,068	4,297	6,936	13,659	17,364	173	<0.001	<0.001	<0.001	<0.001
Zn digestibility	28.1	29.2	34.2	41.6	19.0	2.7	0.181	<0.001	<0.001	0.521
Serum (μg/mL)	1.58	2.58	2.72	2.79	2.74	0.1	<0.001	0.734	<0.001	<0.001
Phase II (d 28)										
Feed (mg/kg DM)	204	730	1,211	2,628	2,699	-	-	-	-	-
Feces (μg/g)	1,172	4,101	7,060	12,866	17,020	121	<0.001	<0.001	<0.001	<0.001
Zn digestibility	32.1	34.2	30.6	44.2	20.1	2.9	0.465	<0.001	<0.001	0.479
Serum (μg/mL)	1.91	3.07	2.85	3.66	3.04	0.11	<0.001	0.249	<0.001	0.169

HME, hot melt extrusion; SEM, standard error of means; DM, dry matter.

1) CL, basal diet without ZnO; HME, basal diet with 500, 1,000, and 2,500 ppm Zn as HME-ZnO; P-ZnO, basal diet with 2,500 ppm Zn as ZnO.

2) Linear (L) and quadratic (Q) effects of HME concentrations.

**Table 6:** Effects of different ZnO sources on intestinal microbial populations (Log10 CFU/g) in weanling pigs (d 28)

Item	CL1)	HME1) (ppm)			P-ZnO1)	SEM	Contrasts2) (p-value)			
	
		500	1,000	2,500			CL vs others	HME vs P-ZnO	L	Q
Ileum										
Total anaerobic bacteria	7.43	7.47	7.54	7.43	7.63	0.07	0.321	0.087	0.849	0.282
*Lactobacillus* spp.	8.27	8.45	8.57	8.41	8.53	0.06	0.019	0.536	0.125	0.041
*Clostridium* spp.	5.59	5.26	5.22	5.3	5.28	0.1	0.015	0.889	0.025	0.019
Coliforms	6.13	5.95	5.89	5.59	5.88	0.06	0.009	0.491	<0.001	0.339
Cecum										
Total anaerobic bacteria	8.65	8.58	8.75	8.66	8.83	0.09	0.657	0.212	0.637	0.904
*Lactobacillus* spp.	8.25	8.32	8.4	8.38	8.45	0.11	0.407	0.609	0.375	0.735
*Clostridium* spp.	6.73	6.65	6.43	6.32	6.39	0.9	0.062	0.581	<0.001	0.887
Coliforms	6.38	6.3	6.18	6.28	6.11	0.14	0.382	0.481	0.495	0.518
Colon										
Total anaerobic bacteria	9.05	9.01	9.22	9.29	9.16	0.11	0.361	0.971	0.051	0.528
*Lactobacillus* spp.	8.05	8.07	8.14	8.32	8.17	0.12	0.431	0.983	0.152	0.538
*Clostridium* spp.	7.23	7.18	7.07	7.01	7.06	0.05	0.007	0.584	0.004	0.95
Coliforms	6.89	6.78	6.67	6.69	6.65	0.08	0.028	0.457	0.081	0.465

HME, hot melt extrusion; SEM, standard error of means.

1) CL, basal diet without ZnO; HME, basal diet with 500, 1,000, and 2,500 ppm Zn as HME-ZnO; P-ZnO, basal diet with 2,500 ppm Zn as ZnO.

2) Linear (L) and quadratic (Q) effects of HME concentrations.

**Table 7:** Effects of different ZnO sources on small intestinal morphology of weanling pigs (d 28)

Item1)	CL1)	HME1) (ppm)			P-ZnO1)	SEM	Contrasts (p-value)2)			
	
		500	1,000	2,500			CL vs others	HME vs P-ZnO	L	Q
Duodenum										
Villus height (μm)	423	447	445	445	454	5.01	<0.001	0.186	0.01	0.029
Crypt depth (μm)	253	247	239	241	243	6.28	0.139	0.913	0.131	0.424
VH/CD	1.68	1.78	1.87	1.85	1.88	0.07	0.008	0.635	0.021	0.112
Jejunum										
Villus height (μm)	394	402	412	405	417	4.86	0.051	0.176	0.156	0.235
Crypt depth (μm)	242	234	224	216	223	8.32	0.067	0.869	0.038	0.964
VH/CD	1.63	1.72	1.85	1.87	1.87	0.06	0.011	0.448	0.011	0.696
Ileum										
Villus height (μm)	228	230	241	259	245	7.88	0.047	0.804	0.006	0.307
Crypt depth (μm)	153	145	140	149	141	7.18	0.251	0.669	0.654	0.268
VH/CD	1.49	1.63	1.77	1.73	1.74	0.11	0.089	0.847	0.144	0.471

HME, hot melt extrusion; SEM, standard error of means; VH/CD, villus height to crypt depth ratio.

1) CL, basal diet without ZnO; HME, basal diet with 500, 1,000, and 2,500 ppm Zn as HME-ZnO; P-ZnO, basal diet with 2,500 ppm Zn as ZnO.

2) Linear (L) and quadratic (Q) effects of HME concentrations.
